# Supplementary material for: Spatiotemporal analysis of medical resource deficiencies in the U.S. under COVID-19 pandemic
Source: PLoS One. 2020 Oct 14;15(10):e0240348. doi: 10.1371/journal.pone.0240348 (PMC7556467; doi:10.1371/journal.pone.0240348)
Supplement: Attachment — Submitted filename: Response to Reviewers.docx [file pone.0240348.s002.docx]

When submitting your revision, we need you to address these additional requirements.

1. Please ensure that your manuscript meets PLOS ONE's style requirements, including those for file naming. The PLOS ONE style templates can be found at

<https://journals.plos.org/plosone/s/file?id=wjVg/PLOSOne_formatting_sample_main_body.pdf> and

<https://journals.plos.org/plosone/s/file?id=ba62/PLOSOne_formatting_sample_title_authors_affiliations.pdf>

**Author response:** Thanks for your comment. We have checked file naming, format, style of the manuscript, and it meets the PLOS ONE’s requirements.

 2.We note that [Figure(s) 3, 4, 5, 6, 8, 9 and 10] in your submission contain [map/satellite] images which may be copyrighted. All PLOS content is published under the Creative Commons Attribution License (CC BY 4.0), which means that the manuscript, images, and Supporting Information files will be freely available online, and any third party is permitted to access, download, copy, distribute, and use these materials in any way, even commercially, with proper attribution. For these reasons, we cannot publish previously copyrighted maps or satellite images created using proprietary data, such as Google software (Google Maps, Street View, and Earth). For more information, see our copyright guidelines: <http://journals.plos.org/plosone/s/licenses-and-copyright>.

 We require you to either (1) present written permission from the copyright holder to publish these figures specifically under the CC BY 4.0 license, or (2) remove the figures from your submission:

 1.    You may seek permission from the original copyright holder of Figure(s) [ 3, 4, 5, 6, 8, 9 and 10] to publish the content specifically under the CC BY 4.0 license.

We recommend that you contact the original copyright holder with the Content Permission Form (<http://journals.plos.org/plosone/s/file?id=7c09/content-permission-form.pdf>) and the following text:

“I request permission for the open-access journal PLOS ONE to publish XXX under the Creative Commons Attribution License (CCAL) CC BY 4.0 (<http://creativecommons.org/licenses/by/4.0/>). Please be aware that this license allows unrestricted use and distribution, even commercially, by third parties. Please reply and provide explicit written permission to publish XXX under a CC BY license and complete the attached form.”

 Please upload the completed Content Permission Form or other proof of granted permissions as an "Other" file with your submission.

In the figure caption of the copyrighted figure, please include the following text: “Reprinted from [ref] under a CC BY license, with permission from [name of publisher], original copyright [original copyright year].”

2.    If you are unable to obtain permission from the original copyright holder to publish these figures under the CC BY 4.0 license or if the copyright holder’s requirements are incompatible with the CC BY 4.0 license, please either i) remove the figure or ii) supply a replacement figure that complies with the CC BY 4.0 license. Please check copyright information on all replacement figures and update the figure caption with source information. If applicable, please specify in the figure caption text when a figure is similar but not identical to the original image and is therefore for illustrative purposes only.

The following resources for replacing copyrighted map figures may be helpful:

USGS National Map Viewer (public domain): <http://viewer.nationalmap.gov/viewer/>

The Gateway to Astronaut Photography of Earth (public domain): <http://eol.jsc.nasa.gov/sseop/clickmap/>

Maps at the CIA (public domain): <https://www.cia.gov/library/publications/the-world-factbook/index.html> and <https://www.cia.gov/library/publications/cia-maps-publications/index.html>

NASA Earth Observatory (public domain): <http://earthobservatory.nasa.gov/>

Landsat: <http://landsat.visibleearth.nasa.gov/>

USGS EROS (Earth Resources Observatory and Science (EROS) Center) (public domain): [http://eros.usgs.gov/#](http://eros.usgs.gov/)

Natural Earth (public domain): <http://www.naturalearthdata.com/>

**Author response:** Thanks for your comment. Figure(s) 3, 4, 5, 6, 8, 9 and 10 were created by authors through ArcGIS and QGIS. So there is no copyright issues in this situation.

Additional Editor Comments:
Do not need to change the article format for your revision.

Reviewers' comments:

Reviewer #1: An estimation of medical resource deficiencies or medical burden at county level is developed using data-driven approach. The developed approach is performed during the COVID-19 pandemic from February 15, 2020 to May 1, 2020 in the U.S. Multiple data sources were used to extract local population, hospital beds, critical care staff, COVID-19 confirmed case numbers, and hospitalization data at county level. The average length of stay from hospitalization data at state level is estimated, and the hospitalized rate at both state and county level are calculated. Then, two medical resource deficiency indices that measure the local medical burden are developed based on the number of accumulated active confirmed cases normalized by local maximum potential medical resources, and the number of hospitalized patients that can be supported per ICU beds per critical care staff, respectively. The medical resources data, and the two medical resource deficiency indices are illustrated in a dynamic spatiotemporal visualization platform based on ArcGIS Pro Dashboards.

The manuscript is well written and structured; however, there are some comments which as follows:

1 – In page 6 – line 108 , it is recommended to move the URL to the references .

**Author response:** Thanks for your comment. This URL is move to the reference list as an expanded item and follows the reference formatting guide.

2 – In page 14 – line 262 , line 267, and line 268, “ MRDI_d” should be in math mode .

**Author response:** All equations have been re-formatted. All “MRDI_d” have been revised to math mode.

3 – in page 15 – line 270 , “ MRDI_d” should be in math mode .

**Author response:** All “MRDI_d” have been revised to math mode.

4 – in page 19 – line 349 , it is recommended to move the URL the references.

**Author response:** Thanks for your comments. This URL in the article is an access link of the dashboard we created and published for audience to use. So we think it is appropriate not to move it to the reference list in this special case.

5 – in page 25 – line 476 , try to replace reference [10] by another reliable source since the arXiv articles are not peer-reviewed .

**Author response:** Thanks for your comment. This reference is accepted as a peer-reviewed paper and the citation is updated in the reference list.

6 – in page 27 – line 505 , try to replace reference [23] by another reliable source since the arXiv articles are not peer-reviewed .

**Author response:** Thanks for your comment. This reference is accepted as a peer-reviewed paper and the citation is updated in the reference list.

7 – in the reference, it is recommended to check the journal guide for authors on how to write URL in the references.

**Author response:** Thanks for your comment, all the reference list is updated based on the guide of PLOS publisher, and follows the page of <https://journals.plos.org/plosone/s/submission-guidelines#loc-references>.

---------------------------

Reviewer #2: The article provide very useful analysis of the health infrastructure in US settings and highlights the capability and quality in terms of the medical equipment and healthcare staff in managing a pandemic such as Covid-19. Although the inferences drawn from the analysis are relevant and important the manuscript can be accepted as short communication or a editorial. The manuscript has limitations in terms of study design and the information presented to be considered as a original research article for publication. It is more like a audit in its present state.

**Author response:** This manuscript is prepared and submitted as part of the Spatiotemporal COVID-19 Rapid Response Collection - FAO: PLOS Collections ([collections@plos.org](mailto:collections@plos.org)). The data were collected and analyzed in a ‘rapid response’ way, providing a practical approach to estimate the possible medical resource deficiencies in the U.S. under COVID-19 pandemic. The original manuscript only described the data and results until May, 2020. In this revised version, we further analyze the data and present the results until August 1, 2020. Based on PLOS editorial policies, the special collection submissions are submitted under the Article Type: “Research Article”.

**--------------------------------------------------------------------------------**

Reviewer #3: Abstract: The manuscript is technically sound and the data generated through synthesis supported the conclusion. However, brief background information was lacked in the very beginning of the abstract. Moreover, the key words would have been placed at the end of the abstract.

**Author response:** Agree. We add background information in the beginning of the abstract: “Coronavirus disease 2019 (COVID‑19) was first identified in December 2019 in Wuhan, China as an infectious disease, and has quickly resulted in an ongoing pandemic.” The key words have been placed at the end of the abstract.

Introduction: The introduction should have been started with a precise statement explaining about pandemic and COVID-19.

**Author response:** Agree. We add background information in the beginning of the introduction: “Coronavirus disease 2019 (COVID‑19) was first identified in December 2019 in Wuhan, China as an infectious disease, and has quickly resulted in an ongoing pandemic.”

Methods: No single figure was presented in the manuscript. I regarded this as a part of the plan by chief and office based editor to minimize bulkiness of the manuscript. The resource-medical interventions compatibility analyses have been performed appropriately and rigorously. All relevant data were included in the manuscript.

**Author response:** All figures were uploaded individually to the PLOS system. Please check the image links or the end of the generated PDF file. There are four figures in Methods section, i.e. Figure 1-4.

Results: Nicely presented and interpreted

**Author response:** Thank you!

Discussion and Conclusions: The findings were discussed and compared. Conclusions were data based.

**Author response:** Thank you!

Reviewer Summary: Minor language edition was made by track changes to bring the manuscript to the level of high standard. All other comments were included by track changes within the manuscript.

**Author response:** Thank you! Most editions are accepted. The only exception is the headings. PLOS ONE’s policy is: “Heading should be written in sentence case (capitalize only the first word of the heading, the first word of the subheading, and any proper nouns and genus names).” So we change the heading accordingly.

----------------------------------------------------------------------------------------------

Reviewer #4: The authors have made a commendable effort in analyzing the medical burden in U.S. on a state level. The graphs and maps included in the manuscript provide a perfect illustration of the conclusions drawn in the study. This study highlights the medical resources deficiency and can help prepare the ground for the future preparedness for the next medical emergency of the century.

**Author response:** Thank you!

---------------------------------------------------------------------------------------

Reviewer #5: The author confined the analysis to four months i.e. from February 2020 to May 2020 whereas COVID 19 get peak in July 2020 in USA. So result about preparedness cannot be realistic without incorporating the peak data.
Discharged patient number should not be consider zero. An estimated value can be consider on the basis of available data to optimize the results.

**Author response:** The manuscript was submitted on May 18, 2020 as part of the Spatiotemporal COVID-19 Rapid Response Collection - FAO: PLOS Collections ([collections@plos.org](mailto:collections@plos.org)). The data were collected and analyzed in a ‘rapid response’ way, providing a practical approach to estimate the possible medical resource deficiencies in the U.S. under COVID-19 pandemic.

In this revised version, we expand our analysis to August 1, 2020. Specifically, we add Fig 9 general MRDI trend on (e) June 15, 2020, and (f) July 15, 2020, and we add daily medical burden MRDI_d_ trend on (b) August 1, 2020. And we revise the related description through the manuscript.

As for the discharged patient number, we still cannot find a reliable data source. We acknowledge this issue in Section 3.1: “It is worth noting that ALOS is likely to be underestimated since we assumed no discharged patients.” And we discuss this issue in Section 5: “It is worth noting that we have not considered the number of discharged patients due to lack of data, leading to a possible slight underestimate of ALOS during the COVID-19 rapid infection period. As a result, $\text{MRDI}_{d}$ may also be slightly underestimated.” By a rough estimation, the MRDI_d_ will be higher around 10% if the discharged patients is 10% within an ALOS, which will not significantly affect the result and visualization effect.

We also list six other possible error sources in Section 5:

(1) “We also did not consider the ratio of ICU patients and acute hospitalized patients due to lack of data, and assumed all hospitalized patients were treated as ICU cases.”
(2) The numbers of registered hospital beds and CCS could be incomplete or not up-to-date;
(3) Critically ill patients in counties without ICU beds and CCS would be sent to neighboring counties with sufficient medical resources;
(4) Some numbers of experienced ICU staff may become ill;
(5) The number of trained professionals may have increased based on emergent recruiting;
(6) The capacity in ICUs and emergency rooms may have been expanded during the crisis.

However, our conclusion is that “$\text{MRDI}_{d}$ can still serve as a useful indicator to measure the county-level medical resource deficiencies, and this index can be improved once more public health data are available in the future.”
